# Reading Self-Efficacy Predicts Word Reading But Not Comprehension in Both Girls and Boys

**DOI:** 10.3389/fpsyg.2016.02056

**Published:** 2017-01-17

**Authors:** Julia M. Carroll, Amy C. Fox

**Affiliations:** ^1^Centre for Research in Psychology, Behaviour and Achievement, Faculty of Health and Life Sciences, Coventry UniversityCoventry, UK; ^2^Department of Psychology, The University of WarwickCoventry, UK

**Keywords:** reading development, self efficacy, reading comprehension, reading motivation, decoding

## Abstract

The relationship between cognitive skills and reading has been well-established. However, the role of motivational factors such as self-efficacy in reading progress is less clear. In particular, it is not clear how self-efficacy relates to word level reading versus comprehension, and whether this differs in boys and girls. This study examines the relationship between self-efficacy, word reading and reading comprehension across the range of reading abilities after controlling for reading-related cognitive factors. One hundred and seventy nine children (86 males and 93 females) between 8 and 11 years old completed a self-report measure of reading self-efficacy together with measures of reading comprehension and word reading, working memory, auditory short-term memory, phonological awareness, and vocabulary. Boys and girls showed similar levels of attainment and reading self-efficacy. Reading self-efficacy was associated with word reading, but not with reading comprehension in either boys or girls. It is argued that this may reflect important differences between reading self-efficacy and more general measures of reading motivation and engagement. Reading self-efficacy is an element of reading motivation that is closely associated with a child’s perceived attainments in reading and is less susceptible to the gender differences seen in broader measures.

## Introduction

There is a growing body of literature on the role of competence beliefs in educational attainment, and a largely independent body of literature on the cognitive predictors of reading. This study aims to combine these two approaches to examine the prediction of word level reading and reading comprehension using a new measure of reading self-efficacy.

Motivation for reading is an important contributory factor to a student’s reading achievement and academic success (Gottfried, 1985). Guthrie and Wigfield (2000) found that when students were highly engaged in reading they comprehended better and their reading outcomes were better. This relationship was mediated by reading behavior: children with high reading engagement showed more reading, which in turn predicted reading comprehension outcomes.

### Self-Efficacy as an Element of Motivation

Self-efficacy refers to peoples’ beliefs that they are capable of carrying out an action to achieve a particular goal (Bandura, 1993). Individuals who perform unsuccessfully may do so, not because they lack the skills and knowledge, but because they lack the efficacy beliefs to use them well (Bandura, 1997). Individuals with high self-efficacy are more likely to persevere in face of difficulties, are more likely to perceive a difficult situation as a challenge, and be less affected by setbacks or failure than individuals with low self-efficacy (Bandura, 1997). Bandura argues that individuals who experience a gradual improvement in their skills over time despite failures experience a sense of mastery, which helps to increase their self-efficacy for a task.

A number of studies have found self-efficacy to be a predictor of students’ academic motivation and learning (Pajares, 1996; Schunk, 1996). Linnenbrink and Pintrich (2003) found that students who had higher levels of self-efficacy were more likely to work harder, persevere and seek support to finish a task. However, it is not a panacea; it has been shown that high self-efficacy without the required knowledge and skills will not result in improved literacy performance (Salomon, 1984; Schunk, 1996), and can result in reduced effort in reading.

Self-efficacy is likely to play a particularly important role in developing reading skills due to the self-teaching mechanism involved in the reading process: successfully deciphering a printed word helps a child to learn to recognize that word automatically in the future (Share, 1995). Therefore these experiences are vital to increasing a child’s reading vocabulary, reading fluency and reading comprehension. A child who tries to read a moderately difficult book and succeeds (raising their self-efficacy) is likely to try to attempt a similar task, reading a book of comparable difficulty in the future (Henk and Melnick, 1995). Wigfield and Guthrie (1997) and Zimmerman (2000) have examined the influence of students’ belief in their own reading abilities and found that students with low reading self-efficacy tried to avoid challenging reading activities and tended to withdraw from tasks they perceived as too difficult.

For the research investigating motivation for academic performance and reading, the terms self-concept, self-worth and self-esteem have been used interchangeably and often confused with self-efficacy. Self-esteem can be defined as an individual’s emotional feelings about their accomplishments (e.g., feeling good or bad about themselves because they can or cannot read a book: Rosenberg et al., 1995). It is distinct from self-concept which relates to an individuals’ general belief about competence (e.g., I am good at reading or maths: Shavelson and Bolus, 1982). Self-concept is formed from past experiences and is heavily influenced by reinforcements and significant evaluations by other people. Bong and Skaalvik (2003) proposed that self-efficacy and self-concept differ in important ways. Self-efficacy comprises of goal referenced, context-specific judgments of competence that are relatively flexible, while self-concept beliefs are hierarchically structured, past-orientated self-perceptions that are relatively stable due to their generality. Because of these differences, self-efficacy beliefs are potentially more changeable in response to intervention. Bong and Skaalvik suggest that self-efficacy acts as a precursor for self-concept development.

### Measuring Reading Self-Efficacy

Research in this area has been made more difficult through the lack of a specific single measure of reading self-efficacy. There have been problems with the measurement of self-efficacy with researchers failing to establish the relationship between self-efficacy and self-esteem and using measures that contain items resembling the concepts of self-concept or self-esteem rather than self-efficacy (Schunk and Pajares, 2002). Many studies have developed their own measures (e.g., Bandura, 1990; Burden, 2000). For example, the Myself As A Learner scale (MALS) is a well-established questionnaire that focuses on academic self-concept (Burden, 2000). Conversely, the Children’s Perceived Self-Efficacy (CPSE) scale (Bandura, 1990) assesses self-efficacy in a wide range of academic, social and leisure contexts only a few questions focus on self-efficacy with regard to literacy and literacy-related skills.

Galla et al. (2014) examine the effect of academic self-efficacy and teacher rated effortful engagement on reading and maths progress over time. They found limited effects of academic self-efficacy on academic outcomes between participants, though within-person changes in self-efficacy predicted growth in reading. The authors suggest that the measure used, the Academic scale of the Self-Efficacy scale for children (Muris, 2001) may have been too broad-based to accurately measure subject-specific self-efficacy.

Similarly, Bruning et al. (2013) assessed three different dimensions of writing self-efficacy (for ideation, writing conventions, and self-regulation) and found that while all three dimensions were associated with self-reported writing grades across the year, self-efficacy for writing conventions was the most strongly related to performance on a standardized writing assessment. This research emphasizes the importance of matching the self-efficacy beliefs to the task undertaken.

This research demonstrates that self-efficacy is highly domain specific and that it is crucial that the domain assessed parallels the self-efficacy beliefs. There is therefore a need for a valid and reliable academic reading self-efficacy questionnaire suitable for use with primary school children. The current research aimed to develop such a measure.

Some studies have shown a positive relationship between self-efficacy and reading (Shell et al., 1995; Solheim, 2011), though this is not consistent (Corkett et al., 2011). Only one of these studies (Solheim, 2011) accounted for reading related cognitive skills, and all have used a different (bespoke in some cases) measure of reading motivation and self-efficacy. Often, these scales have included some items assessing self-concept (e.g., ‘I’m a good reader’: Solheim, 2011) instead of specifically self-efficacy. These issues have made it hard to draw clear conclusions.

### Cognitive Predictors of Reading

Traditionally, the cognitive predictors of reading are examined separately from socio-emotional predictors, but it is important to know how the different predictors overlap. The cognitive predictors of learning to read may vary across educational systems (Cunningham and Carroll, 2011). However, in children learning to read in English and receiving phonics based tuition, a few relatively short measures of cognitive processing provide good predictors of a child’s reading progress (Wagner et al., 1994; Muter et al., 1998), though the value of these predictors may reduce with age (Kirby et al., 2003). For children who are taught using phonics-based systems, these generally include code related skills such as letter knowledge and phonological awareness for word reading, and broader skills such as vocabulary and short-term memory (STM) for reading comprehension (Wagner et al., 1997). It is likely that these skills are associated with the self-teaching strategies suggested by Share (1995), as well as with self-efficacy itself. If a child is taught using a phonics-based system and has good phonological awareness for example, they are more likely to be able to sound out an unknown word effectively. They are also more likely to believe that they can sound out words effectively – in other words, to show high self-efficacy in this area. For this reason, a child’s self-efficacy may be explained largely in terms of their underlying cognitive abilities. In other words, children may show accurate self-perceptions of their abilities, and this may account for the links between self-efficacy and reading. In order to minimize this explanation of results, we included in our test battery a range of cognitive abilities well-known to predict literacy progress.

### Contrasting Reading Accuracy versus Reading Comprehension

Studies of the role of motivation and competence beliefs have typically focused on reading comprehension as an outcome measure, while cognitive approaches have normally differentiated word level reading and reading comprehension. There is evidence of dissociation between the two skills, with children often showing weaknesses in one, but not both, of the two skills. Individuals with poor word level reading can be labeled as dyslexic. Perhaps surprisingly, children with dyslexia often, though not always, show relatively good comprehension of what they read. Those with specific comprehension difficulties are labeled poor comprehenders (Nation and Snowling, 1998). These children show good word-level reading, but poor understanding of what they read. These two profiles are associated with different cognitive weaknesses. It is also of interest to know whether word reading and reading comprehension have different relationships with self-efficacy.

### Gender Differences in Reading Motivation and Engagement

There is growing evidence that boys and girls show different relationships between self-beliefs and academic attainments, with the strongest evidence pertaining to engagement. For boys, engagement is closely related to reading achievement, while for girls, reading engagement is a less important predictor (Logan and Medford, 2011). To our knowledge, this relationship has not been examined with respect to reading self-efficacy. A recent meta-analysis (Huang, 2013) found relatively small gender differences in self-efficacy beliefs in the language arts. In contrast to other academic disciplines, females had higher self efficacy beliefs than males, but gender differences only started to appear in late adolescence and adulthood. However, this small gender difference does not preclude the possibility that the links between self-efficacy and reading achievement may differ in boys and girls. Other recent research has indicated different predictors of reading outcome in boys and girls with respect to visual processing (Huestegge et al., 2012).

In summary, there is support for a relationship between self-efficacy and reading, but current studies have focused on reading comprehension rather than word level reading, have used a variety of self-efficacy measures and have not consistently taken into account other reading related cognitive variables. Using this questionnaire we will investigate the following hypotheses:

(1) Reading self-efficacy will be associated with higher scores in word reading and reading comprehension.
(2) Reading self-efficacy levels will be similar in boys and girls, but reading self-efficacy will be more closely related to reading boys.
(3) Reading self-efficacy will predict variance in word reading and reading comprehension after controlling for reading-related cognitive variables (phonological awareness, vocabulary, and STM).

## Materials and Methods

The project was approved by the University of Warwick Humanities and Social Sciences ethics committee prior to commencing.

### Participants

One hundred and seventy-nine children from years 4, 5, and 6 (86 males and 93 females, aged between 8.05 and 11.05 years, *M* = 9.08 years) from two primary schools in the Central England took part in the study. Both schools followed a state-mandated phonics based system for teaching literacy. One class per year group was randomly selected at each school. Each class contained children with a range of reading abilities. Both schools served predominately white middle class families with English as their first language. Consent was gained from the caregiver for all children prior to participation.

### Measures

#### Reading Self-Efficacy Questionnaire

A 20 items self-report questionnaire was designed to assess children’s reading self-efficacy. The questionnaire attempted to address issues of developing self-report scales for children (Fulmer and Frijters, 2009). Participants were asked to rate the strength of their belief that they could carry out a range of reading-related tasks or reading challenges on a scale of 1 to 7. The items were developed to reflect graded levels of reading task demands and the language used was kept at basic level to aid comprehension. Practice items were developed from the items used in Bandura’s (1990) Children’s Self-Efficacy Scale. The overall score was the mean rating given (after reverse scoring appropriate items) and therefore could range between 1 and 7. The questionnaire is presented in Supplementary Material.

#### Pilot Testing

Thirty children between eight and 10 years old completed the 20 items questionnaire in a one-to-one situation with the experimenter. One item was removed due to difficulties in children understanding it. Overall, the questionnaire demonstrated good internal reliability (α = 0.912). The inter-item correlation coefficients for the items were all positive (0.38 to 0.78). In addition, verbal feedback from the children indicated that a number of participants had difficulty understanding the meaning of the following terms: ‘reading material,’ ‘read aloud,’ and ‘recognize.’ Therefore, to aid questionnaire completion and to present the items that were more understandable, item 1 was changed from *“read aloud in class”* to *“read out loud in front of the class,”* item 10 was changed from *“recognize words easily when I read”* to *“make out words easily when I read”* and item 17 was changed from *“read difficult material”* to *“read difficult books.”* In addition to this, Item 6 was changed from “*read things that are harder than I normally read”* to *“read things that are harder than the books I normally read at school*” in an effort to make the question more specific, based on informal feedback from teachers.

### Reading Measures

#### Reading Comprehension

Reading comprehension was assessed using a shortened version of Vernon-Warden Reading Test – revised (Hedderley, 1996). In this test children were given 10 min to identify the correct word from a range of options that completes a sentence (e.g., ducks swim in a ___: bucket, pond, yard, garden). The items increased in difficulty through the questionnaire. The final six items, which are the most difficult and aimed at secondary school students, were omitted, leaving 34 questions. Each item scored 1 point, therefore the maximum score was 34. The inter-item reliability of this shorter measure was α = 0.73.

#### Word Level Reading

The Test of Word Reading Efficiency (TOWRE) by Torgeson et al. (1997) measured ability to pronounce printed words and non-words accurately and fluently using two subtests. Phonemic decoding is measured by asking children to read as many nonsense words as possible in 45 s. Word reading fluency is measured by asking children to read as many words as possible in 45 s. The test provides individual and total standard scores. Reliability reported in the manual for total word reading is α = 0.96.

### Cognitive Measures

#### Short-Term Auditory Memory (STM)

The Recall of Digits Forward subtest from the British Ability Scale second edition (BAS II; Elliot et al., 1997) was used to assess STM. The test was administered according to the standard procedure. Participants heard lists of digits increasing in length and were asked to repeat these back to the experimenter in order. T-scores (age standardized scores with a mean of 50 and standard deviation of 10) are reported.

#### Working Memory

Working memory was assessed using the Recall of Digits Backward subtest from the BAS II (Elliot et al., 1997). Participants heard lists of digits increasing in length and were asked to repeat these back to the experimenter in a backward order. T-scores (age standardized scores with a mean of 50 and standard deviation of 10) are reported.

#### Vocabulary

Expressive language was assessed using the Word Definitions subtest of the BAS II (Elliot et al., 1997). Participants are given increasingly complex spoken words to describe or define. T-scores (age standardized scores with a mean of 50 and standard deviation of 10) are reported.

#### Phonological Awareness

The Spoonerisms test from the Phonological Assessment Battery (Frederickson et al., 1997) was used to assess children’s ability to segment single syllable words and then synthesize the segments to provide new words or word combinations. The test consists of two parts. In part 1 the child is asked to replace the first sound of a word with a new sound. In the second part the child is asked to exchange the initial sounds of two words. Each part is given a time limit of 3 min, and the maximum score is 30. Standard scores (age standardized scores with a mean of 100 and standard deviation of 15) are reported.

### Procedure

Participants took part in three sessions. In the first session the reading self-efficacy questionnaire was administered to the whole class at the start of the lesson. This session took approximately 15 min. Any participants who were absent at the time completed the questionnaire as a small group at a later date. Once the practice items were completed participants then worked on the reading self-efficacy questionnaire. Participants were read the instructions at the top of the page aloud to remind them to consider each of the items and to rate them in terms of how certain they could carry out the reading actions described in the questionnaire. They were instructed to consider reading in terms of things they read in books, magazines, newspapers, comics, emails or on the internet and were told to work individually. It was emphasized that they should seek help from the experimenter if there were any words or items that they did not understand. Participants were asked to indicate when they had completed the questionnaire and to wait in silence until the whole class had finished. Children who had difficulties in reading the items were given extra support by the experimenter, teacher or teaching assistant who read aloud the items.

In the second session participants completed the Vernon-Warden Reading Test – revised (Hedderley, 1996) in small groups. They completed the practice example with the experimenter and were given 10 min to complete the 34 items.

In the third session participants were tested individually for 20 min in a quiet area and completed tests of working memory, STM, vocabulary, phonological awareness, and word reading ability in a fixed order.

## Results

### Preliminary Data Analysis

The reading self-efficacy questionnaire demonstrated acceptable inter-item reliability for the current study, with a Cronbach alpha coefficient of 0.89. Four children were outliers, with scores more than 3 standard deviations below the mean. Three of these children demonstrated very low reading scores, while one had reading within the average range. These four children were excluded from further analyses.

### Gender Differences in the Tasks

The descriptive statistics for each of the measures used appear in **Table 1**, together with independent samples *t*-test comparing the two genders. There were no significant differences between males and females in any of these scores. The association between age and each measure for males and females is shown in **Table 2**. Perhaps surprisingly, there is a small but significant association between reading self-efficacy and age. Older children had slightly higher reading self-efficacy, perhaps because they are more able to do the specific reading related tasks detailed in the questionnaire. To account for the association between age and cognitive measures, age is included as a control variable in future analyses.

**Table 1:** Mean scores (standard deviations in parentheses) for males and females in self-efficacy, reading, and cognitive measures.

Task	*Males (N = 83)*	*Females (N = 91)*	*T-test*
Reading self-efficacy	5.86 (0.78)	5.85 (0.72)	*t*(172) = 0.15, *p* = 0.88
Reading comprehension (maximum score of 34)	12.77 (4.72)	13.87 (5.25)	*t*(172) = -1.45, *p* = 0.15
Word reading (TOWRE standard score)	112.14 (15.80)	110.00 (15.04)	*t*(172) = 0.92, *p* = 0.36
STM (T-score)	54.80 (7.64)	56.08 (8.67)	*t*(172) = -1.03, *p* = 0.30
Working memory (T-score)	50.89 (7.97)	51.44 (9.78)	*t*(172) = -0.40, *p* = 0.69
Vocabulary (T-score)	47.02 (10.21)	46.00 (10.02)	*t*(171) = 0.67, *p* = 0.51
Phonological awareness (standard score)	102.51 (11.76)	102.56 (12.58)	*t*(172) = 0.92, *p* = 0.36

**Table 2:** Correlations between age and reading and cognitive measures in males and females.

	*Males* (*N* = 83)	*Females* (*N* = 91)
(1) Reading self-efficacy	0.21 (*p* = 0.06)	**0.26 (*p* = 0.01)**
(2) Reading comprehension	**0.43 (*p* < 0.01)**	**0.41 (*p* < 0.01)**
(3) Word reading (TOWRE)	**0.38 (*p* < 0.01)**	**0.42 (*p* < 0.01)**
(4) Digit span forward	**0.29 (*p* = 0.01)**	**0.34 (*p* < 0.01)**
(5) Digit span backward	**0.40 (*p* < 0.01)**	**0.34 (*p* < 0.01)**
(6) Vocabulary	**0.26 (*p* = 0.02)**	**0.40 (*p* < 0.01)**
(7) Phonological awareness	**0.34 (*p* < 0.01)**	**0.34 (*p* < 0.01)**

*p-values are shown in parentheses. Bold indicates that the correlation is significant at *p* < 0.05.*

### Relationships between Variables

Partial correlations controlling for age were performed to determine the relationship between reading self-efficacy, reading performance and the cognitive measures separately for the male and female participants. The correlations can be seen in **Table 3**. A linear step-up analysis (Benjamini et al., 2006) suggests that the significance level should be set at *p* < 0.03. Overall, correlations were very similar across males and females. Reading self-efficacy was significantly positively correlated with word reading, but not with reading comprehension. As expected the cognitive measures correlated with the reading performance measures and amongst themselves.

**Table 3:** Correlations between reading self-efficacy, reading performance, and cognitive measures [males (*N* = 83) below the diagonal and females (*N* = 91) above the diagonal] after controlling for age.

	*1*	*2*	*3*	*4*	*5*	*6*	*7*
(1) Reading self-efficacy (mean score)	-	0.19; 0.08	**0.41; <0.01**	0.23; 0.03	**0.24; 0.02**	**0.30; <0.01**	**0.37; <0.01**
(2) Reading comprehension (total score)	0.17; 0.12	-	**0.44; <0.01**	0.29; 0.03	**0.36; <0.01**	**0.40; <0.01**	**0.47; <0.01**
(3) Word reading (TOWRE)	**0.39; <0.01**	**0.41; <0.01**	-	**0.38; <0.01**	**0.44; <0.01**	**0.39; <0.01**	**0.54; <0.01**
(4) Digit span forward	0.12; 0.30	**0.29; 0.01**	**0.32; <0.01**	-	**0.51; <0.01**	**0.27; <0.01**	**0.34; <0.01**
(5) Digit span backward	**0.32; <0.01**	**0.32; <0.01**	**0.39; <0.01**	**0.43; <0.01**	-	0.22; 0.04	**0.34; <0.01**
(6) Vocabulary	0.24; 0.03	**0.55; <0.01**	**0.36; <0.01**	0.20; 0.08	**0.29; <0.01**	**-**	**0.52; <0.01**
(7) Phonological awareness	**0.33; <0.01**	**0.40; <0.01**	**0.50; <0.01**	**0.32; <0.01**	**0.55; <0.01**	**0.42; <0.01**	-

*p-values are shown under *r-*values. *p*-values are shown following the semi colon for all correlations where *p* < 0.05. Correlations for which *p* < 0.01 are shown in bold.*

### Does Reading Self-Efficacy Predict Reading Performance after Accounting for Cognitive Measures?

To determine the contribution of reading self-efficacy in predicting word reading after accounting for the contribution of cognitive variables (working memory, vocabulary, auditory STM, and phonological awareness) two hierarchical multiple linear regressions were computed separately for males and females. School was entered into the first block, age was entered in the second block, the cognitive measures were entered into the third block and mean reading self-efficacy score into the fourth block, to examine the role of self-efficacy after accounting for cognitive variables. These analyses are presented in **Tables 4** and **5**. For both females (**Table 4**) and males (**Table 5**), reading self-efficacy was a small but significant predictor of reading skills after controlling for cognitive predictors. In order to examine whether the role of self-efficacy differed significantly across males and females, a regression analysis for the full sample was carried out, including gender as a dummy variable and the interaction between gender and self-efficacy at the final step. This is shown in **Table 6**. The interaction term did not account for significant additional variance (β = -0.11, *p* = 0.82).

**Table 4:** Regression analysis predicting word reading using cognitive measures and reading self-efficacy in females.

Variable	*B*	SE *B*	β	*t*	*R*^2^ change
Block 1: School	-2.83	2.42	-0.12	-1.17	0.02
Block 2: Age	0.46	0.10	0.43	4.45^∗∗^	0.18
Block 3:					0.31
STM	0.04	0.06	0.07	0.74	
Working memory	0.13	0.05	0.25	2.47^∗^	
Vocabulary	0.08	0.06	0.13	1.27	
Phonological awareness	0.56	0.16	0.36	3.59^∗∗^	
Block 3: Reading self-efficacy	2.76	1.36	0.18	2.03^∗^	0.02

*^∗^*p* < 0.05, ^∗^*^∗^p* < 0.01.*

**Table 5:** Regression analysis predicting word reading using cognitive measures and reading self-efficacy in males.

Variable	*B*	SE *B*	β	*t*	*R*^2^ change
Block 1: School	-2.68	2.33	-0.13	-1.15	0.02
Block 2: Age in months	0.38	0.10	0.37	3.63^∗∗^	0.14
Block 3:					0.25
STM	0.09	0.07	0.13	1.24	
Working memory	0.07	0.07	0.11	0.91	
Vocabulary	0.11	0.07	0.19	1.62	
Phonological awareness	0.49	0.19	0.31	2.58^∗^	
Block 3: Reading self-efficacy	2.87	1.30	0.21	2.21^∗^	0.04

*^∗^*p* < 0.05, ^∗^*^∗^p* < 0.01.*

**Table 6:** Regression analysis predicting word reading using cognitive variables and reading self-efficacy in the full sample.

Variable	*B*	SE *B*	β	*t*	*R*^2^ change
Block 1: School	-2.79	1.67	-0.13	-1.67	0.02
Block 2: Age in months	0.42	0.07	0.40	5.78^∗∗^	0.16
Block 3: Gender	0.32	1.54	0.01	0.21	<0.01
Block 4:					0.28
STM	0.07	0.04	0.11	1.48	
Working memory	0.10	0.04	0.19	2.48^∗^	
Vocabulary	0.10	0.04	0.16	2.14^∗^	
Phonological awareness	0.51	0.12	0.33	4.37^∗∗^	
Block 5: Reading self-efficacy	2.75	0.92	0.19	2.99^∗∗^	0.03
Block 6: Gender by self efficacy interaction	-0.39	1.67	-0.11	-0.23	<0.01

*^∗^*p* < 0.05, ^∗^*^∗^p* < 0.01.*

In order to predict reading comprehension, the predictor variables were the same and they were entered in the same order, with the exception that word reading was entered at block 3, before the cognitive predictor variables. These are shown in **Tables 7** and **8**. For both genders, school, age and word reading level accounted for a significant proportion of the variance. While the cognitive predictors were significant additional predictors as a whole, no single cognitive variable was a significant predictor for females, and only vocabulary was marginally significant for males. For both genders, reading self-efficacy was not a significant predictor of reading comprehension after the cognitive variables were controlled.

**Table 7:** Regression analysis predicting reading comprehension using cognitive variables and reading self-efficacy in females.

Variable	*B*	SE *B*	β	*t*	Total *R*^2^ change
Block 1: School	-4.18	1.02	-0.40	-4.09^∗∗^	0.16
Block 2: Age in months	0.20	0.04	0.40	4.53^∗∗^	0.16
Block 3: Word reading	0.19	0.04	0.41	4.60^∗∗^	0.14
Block 4:					0.05
STM	0.02	0.03	0.08	0.74	
Working memory	0.01	0.03	0.06	0.56	
Vocabulary	0.01	0.03	0.05	0.47	
Phonological awareness	0.01	0.08	0.20	1.81	
Block 5: Reading self-efficacy	-0.44	0.66	-0.06	-0.66	<0.01

*^∗^*p* < 0.05, ^∗∗^*p* < 0.01.*

**Table 8:** Regression analysis predicting reading comprehension using cognitive variables and reading self-efficacy in males.

Variable	*B*	SE *B*	β	*t*	Total *R*^2^ change
Block 1: School	-3.59	0.96	-0.38	-3.73^∗∗^	0.15
Block 2: Age in months	0.19	0.04	0.42	4.51^∗∗^	0.17
Block 3: Word reading	0.16	0.04	0.35	3.80^∗∗^	0.11
Block 4: STM	0.04	0.03	0.13	1.39	0.09
Working memory	<-0.01	0.03	-0.02	-0.13	
Vocabulary	0.08	0.03	0.29	2.65^∗^	
Phonological awareness	0.07	0.08	0.10	0.90	
Block 4: Reading self-efficacy	-0.35	0.56	-0.06	-0.62	<0.01

*^∗^*p* < 0.05, ^∗^*^∗^p* < 0.01.*

## Discussion

The associations between reading performance, self-efficacy and cognitive skills were examined. Our hypotheses received partial support. In line with the first and third hypothesis, reading self-efficacy was associated with word reading, and was in fact a statistically significant predictor of overall reading performance after taking into account cognitive reading-related factors of working memory, vocabulary, and phonological awareness. Boys and girls showed similar levels of reading self-efficacy. However, the reading self-efficacy measure was not significantly associated with reading comprehension.

When the reading measures were considered independently, reading self-efficacy predicted reading fluency but not reading comprehension. For word reading, working memory, and phonological awareness were significant predictors, while school, vocabulary, and STM were not. For reading comprehension, school and word reading were significant predictors, and vocabulary was a significant predictor in boys. For both reading measures STM was not a significant predictor of reading.

This research expands on previous studies investigating self-efficacy and reading by controlling for relevant cognitive skills. We have shown a clear and specific relationship between reading self-efficacy and word level reading skill.

### Gender and Age Differences in Reading Self-Efficacy

In line with previous research (Huang, 2013), we showed similar levels of reading self-efficacy in males and females. There was also no indication of a significantly different relationship between self-efficacy and reading in males and females. This contrasts with patterns shown for reading engagement in other research (Logan and Medford, 2011). There was, however, a significant association between age and self-efficacy, with older children showing greater self-efficacy. This contrasts with previous research: for example, Smith et al. (2012) found a drop in both reading motivation and self-efficacy between 8 and 12 years of age in a New Zealand sample, despite significant increases in reading skill. Further research is needed to elucidate this relationship.

Self-efficacy for a particular behavior (e.g., reading) is likely to affect how individuals perform on tasks involving this behavior (Bandura, 1997). A child with high self-efficacy is more likely to work harder and persist longer than a child who doubts his or her capabilities (Linnenbrink and Pintrich, 2003). Individuals who believe they can be successful in an activity are more likely to engage in it (Zimmerman, 1995; Bandura, 1997; Schunk and Zimmerman, 1997). Therefore, applying the theory to the current findings, it may be that self-efficacy judgments affect an individual’s response to reading through the amount of effort expended during reading, involvement in reading activities, choice of reading activities, effort expended during comprehension and therefore overall reading achievement (Henk and Melnick, 1995). However, some caution must be attached to these conclusions. It is also important not to overlook alternative interpretations of the relationship. Children with good reading skills are likely to be aware that they are good at reading, and therefore have a higher self-efficacy. There is likely to be a bidirectional relationship between these variables that unfortunately cannot be teased out in a cross-sectional study (Morgan and Fuchs, 2007). Furthermore, high reading self-efficacy does not automatically result in reading improvements. It is unlikely to lead to improvements if the underlying cognitive skills are not already present (Schunk, 1996). It is also important to be aware that these children were taught using a phonics based system and results are likely to differ according to the teaching they have received (Thompson et al., 2009).

### The Role of Self-Efficacy in Reading Accuracy versus Reading Comprehension

We have established that reading self-efficacy accounts for variation in word level reading once cognitive skills have been taken into account. However, this relationship was not seen for reading comprehension. There are multiple possible reasons for this finding. First, it may be that children of this age group are less aware of, or less focused upon, their comprehension skills in comparison to their decoding skills. Further, the questions in the self-efficacy measure mentioned reading in general terms and activities such as working out unknown words, but did not focus on understanding what the child had read. It may well be that these children placed less emphasis on these skills.

However, this finding was in opposition to results of a similar study by Solheim (2011). The results may have differed from Solheim’s study in terms of the tasks used. Our measure of comprehension showed only adequate reliability and was not predicted by vocabulary, in contrast to previous studies (e.g., Joshi, 2005; Ricketts et al., 2007). Therefore, one possible explanation for the lack of association between these variables is that the reading comprehension task used lacked validity. In light of this, future studies may do well to use alternative reading comprehension measures.

### Educational Implications

One area that has not been fully explored in the literature is the relationship between self-efficacy and reading for those receiving support with reading though extra tuition or other educational intervention. One might expect that children with high-self efficacy would show a better response to reading interventions. It is known that a significant subgroup of children show a lack of responsiveness even to high quality, well-structured phonics based intervention (Torgesen, 2000). Reading self-efficacy could be a significant factor in children’s lack of responsiveness to intervention. However, the reverse might also be true, in that a good quality reading intervention may improve a child’s reading self-efficacy, and in turn that would help to improve their reading skills. Future research could examine whether reading self-efficacy for these children changes over the course of an intervention.

On the basis of these results it seems that self-efficacy is associated with reading performance and reading fluency, regardless of cognitive abilities. When teaching reading skills it may also be helpful to emphasize the development of children’s reading self-efficacy. While ways to improve self-efficacy are currently under-researched, they are likely to include repeated successful encounters with print. Hence, children who receive regular practice at reading within their capabilities are likely to increase in self-efficacy over time (Bandura, 1997; Guthrie and Wigfield, 2000). This contrasts with the experience of many children with reading difficulties, who will often be given reading tasks beyond their capabilities, and highlights the benefits in consolidating existing learning.

## Ethics Statement

Humanities and Social Sciences Research Ethics Committee, University of Warwick. Written consent was sought from the headteachers of the schools involved and from the parents of the children involved. Consent was sought from parents and guardians of the children tested. Children were also told they could choose not to take part in the research at any point.

## Author Contributions

Both authors developed the idea for the project together. AF developed the self efficacy scale and carried out the testing with assistance from Rachel Dennan. Both JC and AF contributed to the analysis and write up.

## Conflict of Interest Statement

The authors declare that the research was conducted in the absence of any commercial or financial relationships that could be construed as a potential conflict of interest.
