# Supplementary material for: Reading Self-Efficacy Predicts Word Reading But Not Comprehension in Both Girls and Boys
Source: Front Psychol. 2017 Jan 17;7:2056. doi: 10.3389/fpsyg.2016.02056 (PMC5239817; doi:10.3389/fpsyg.2016.02056)
Supplement: Supplementary file 1 [file Data_Sheet_1.docx]

**Appendix**

**Reading self-efficacy questionnaire**

**Practice**

Read each item and rate how certain YOU are that you can do the actions described below by circling one of the numbers below the item. High scores equal a higher certainty that you can do the action. Please wait for instructions before starting the practice items.

**Lift a bag of sugar**

Very certain I cannot do- 1 2 3 4 5 6 7 - Very certain I can do

**Lift one of my class friends**

Very certain I cannot do- 1 2 3 4 5 6 7 - Very certain I can do

**Lift two of my class friends**

Very certain I cannot do- 1 2 3 4 5 6 7 - Very certain I can do

**Main questionnaire**

Read each sentence and rate how certain **YOU** are that you can do the things described below. It is important you tell us what **YOU** think about your reading. When you think about reading, think about the any reading that you do at school and at home. These could be things you read in books, magazines, newspapers, comics, emails, text messages and the internet. To give an answer circle one of the numbers on the scale below the item. If

Very certain I cannot do - 1 2 3 4 5 6 7 - Very certain I can do

1. Read out loud in front of the class
2. Continue reading even when I find it difficult
3. Work out the sounds in words I have not seen before
4. Sound out a word that I find hard to read
5. Read on my own without an adult’s help
6. Read things that are harder than the book I normally read at school
7. Know what I can do to improve my reading
8. Continue reading even when I find the subject boring
9. Read out loud quickly and still get words right
10. Make out words easily when I read
11. Improve my reading if I really want to
12. Continue reading even when I do not like the subject
13. Read as well as my friends
14. Continue reading even when I get frustrated
15. Practice reading in my spare time even when I don’t have to
16. Read without making lots of mistakes
17. Read difficult books
18. Read a book I have not read before
19. Work out the sounds in words I have not seen before
